# Exploratory Analysis of Selected MicroRNAs as Tissue-Based Candidate Biomarkers in Ovarian Cancer

**DOI:** 10.3390/ijms27209122

**Published:** 2026-10-15

**Authors:** Mateusz Kozłowski, Dominika Borzyszkowska, Michał Lubkowski, Nina Komaniecka, Aleksandra Mikulka, Radosław Birger, Piotr Kolczewski, Agnieszka Brodowska, Mateusz Kurzawski, Agnieszka Kempinska-Podhorodecka, Aneta Cymbaluk-Płoska

**Affiliations:** 1 Department of Gynecological Surgery and Gynecological Oncology of Adults and Adolescents, Pomeranian Medical University in Szczecin, Al. Powstańców Wielkopolskich 72, 70-111 Szczecin, Poland; 2 Department of Pediatric Surgery, Orthopedics and Urology with a Subdivision of the Burn Treatment, Independent Public Specialist Healthcare Facility “Zdroje”, 70-780 Szczecin, Poland; 3 Department of Functional Anatomy, Pomeranian Medical University in Szczecin, Al. Powstańców Wielkopolskich 72, 70-111 Szczecin, Poland; michal.lubkowski@pum.edu.pl (M.L.); agnieszka.kempinska.podhorodecka@pum.edu.pl (A.K.-P.); 4 Department of Experimental and Clinical Pharmacology, Pomeranian Medical University in Szczecin, Al. Powstańców Wielkopolskich 72, 70-111 Szczecin, Poland; 5 Department of Gynecologic Oncology and Reconstructive Gynecology, Department of Obstetrics and Gynecology with the High-Risk Pregnancy Unit, Independent Public Specialist Healthcare Facility “Zdroje”, 70-780 Szczecin, Poland; 6 Laboratory of Pharmacodynamics, Pomeranian Medical University in Szczecin, Plac Polskiego Czerwonego Krzyża 1, 71-244 Szczecin, Poland; radoslaw.birger@pum.edu.pl (R.B.);; 7 Private Clinic Esthegyn, ul. Mazurska 7, 70-424 Szczecin, Poland; 8 Department of Gynecology, Endocrinology and Gynecological Oncology, Pomeranian Medical University in Szczecin, Unii Lubelskiej 1, 71-252 Szczecin, Poland

**Keywords:** ovarian cancer, microRNAs, biomarkers, diagnosis, gene expression, ROC curve, survival analysis, miR-425-5p, miR-381-3p, miR-582-5p

## Abstract

The aim of the study was to evaluate the expression and exploratory diagnostic potential of selected microRNAs (miRNAs) in ovarian cancer tissue compared with benign ovarian lesions. Tissue samples from 24 patients with epithelial ovarian cancer and 22 patients with benign ovarian lesions were analyzed by quantitative real-time polymerase chain reaction. Ten candidate miRNAs were assessed; six met the predefined detection criteria and were retained for analysis. Group differences were tested with non-parametric methods and false discovery rate (FDR) correction. Diagnostic performance was evaluated by receiver operating characteristic (ROC) analysis, and multivariable logistic regression adjusted for age and menopausal status. Survival analyses were exploratory. miR-381-3p was downregulated, whereas miR-582-5p and miR-425-5p were upregulated in ovarian cancer compared with benign ovarian lesions; all three differences remained significant after FDR correction. miR-425-5p showed the highest individual diagnostic accuracy (AUC = 0.854) and remained associated with ovarian cancer after adjustment for age and menopausal status (OR = 7.84; 95% CI = 2.05–29.97; *p* = 0.003). This association was also retained in Firth penalized logistic regression. An exploratory three-miRNA panel achieved an apparent AUC of 0.858 but did not significantly outperform miR-425-5p alone (DeLong *p* = 0.596); its optimism-corrected bootstrap AUC was 0.822. No significant associations with survival were identified. miR-425-5p was the strongest candidate biomarker in this exploratory tissue-based study. Independent validation is required before clinical application.

## 1. Introduction

Ovarian cancer remains one of the leading causes of gynecologic cancer mortality, largely because clinical symptoms are often nonspecific and many patients are diagnosed at an advanced stage [1,2]. Despite improvements in cytoreductive surgery, platinum-based chemotherapy, and targeted treatment, outcomes remain unsatisfactory for a substantial proportion of patients. Serum cancer antigen 125 (CA125) and human epididymis protein 4 (HE4) are widely used in clinical assessment, but neither provides sufficient sensitivity and specificity for population screening or reliable detection of all early-stage tumors [3,4]. The identification of complementary molecular biomarkers therefore remains an important research objective.

MicroRNAs (miRNAs) are short non-coding RNAs that regulate gene expression at the post-transcriptional level through interaction with target messenger RNAs [5]. They participate in proliferation, differentiation, apoptosis, stress responses, migration, and other processes central to carcinogenesis [6,7]. Aberrant miRNA expression has been described throughout ovarian tumor initiation, progression, metastasis, and treatment resistance [8]. Their relative stability in tissue and body fluids and their close relationship to cancer-associated signaling pathways make them attractive biomarker candidates [7]. However, the biological effect of an individual miRNA is strongly context dependent, and promising signals identified in small cohorts require careful validation before clinical translation.

The candidate miRNAs evaluated in this study were selected on the basis of published evidence linking them to cancer-related pathways while leaving their expression and clinical relevance in ovarian cancer incompletely characterized. miR-425-5p has been associated with proliferative PI3K/AKT signaling [9]; miR-582-5p can regulate NOTCH1 signaling [10]; miR-574-5p has been linked to Wnt/β-catenin-related tumor progression [11]; and miR-664 has demonstrated tumor-suppressive activity in other malignancies [12]. Our previous review also highlighted that many proposed ovarian-cancer miRNAs have been evaluated only in individual or heterogeneous studies and require further validation [13]. Accordingly, the present work was conceived as a targeted candidate-miRNA study rather than an unbiased discovery experiment.

A tissue-based approach can establish whether a candidate is dysregulated within the tumor before blood-based validation. However, tissue expression is not equivalent to a screening biomarker because release into circulation, extracellular-vesicle packaging, stability, and pre-analytical handling may alter signals in serum or plasma. The present analysis therefore addresses discrimination in resected tissue and should be viewed as a step toward, rather than evidence of, non-invasive implementation [7,8,13].

We therefore evaluated the expression of ten selected miRNAs in ovarian cancer tissue and benign ovarian lesions. After quality and detection filtering, six miRNAs were retained for the final analyses. We examined their relationships with disease status and clinicopathological characteristics, assessed exploratory diagnostic performance, and investigated possible prognostic associations. The principal aim was to identify biologically plausible candidate signals for subsequent validation rather than to establish a clinically deployable diagnostic test.

## 2. Results

### 2.1. Patient Characteristics

Patients with ovarian cancer were older than controls (median 67.1 vs. 48.3 years; *p* = 0.02) and more often postmenopausal (75.0% vs. 45.5%; *p* = 0.04). BMI did not differ significantly. A comparison of the clinicodemographic characteristics of the study and control groups, as well as the clinicopathological characteristics of the study group, is presented in Table 1. The control group included functional ovarian cysts (*n* = 8), simple ovarian cysts (*n* = 7), and benign serous epithelial ovarian tumors (*n* = 7).

### 2.2. Differential miRNA Expression

Among the six detectable miRNAs, miR-381-3p was lower in ovarian cancer (*p* = 0.002), whereas miR-582-5p (*p* = 0.003) and miR-425-5p (*p* < 0.001) were higher (Table 2). These three cancer-control associations remained significant after FDR correction (all q = 0.030). miR-574-5p differed by menopausal status and also remained significant after FDR correction (q = 0.030). Other subgroup findings did not survive multiplicity adjustment. Complete subgroup comparisons and FDR results are provided in Supplementary Tables S1 and S2.

The direction of the three cancer-associated expression changes was internally consistent with the subsequent ROC analyses: miR-425-5p and miR-582-5p were higher in malignant tissue, whereas miR-381-3p was lower. The complete clinicopathological subgroup analysis, including FIGO stage, BMI, menopausal status, and histological subtype, is provided in Supplementary Table S2. After correction across the predefined comparison family, the cancer-control differences for miR-425-5p, miR-381-3p, and miR-582-5p remained significant, together with the association between miR-574-5p and menopausal status (Supplementary Table S1). Thus, the principal cancer-control findings were not dependent solely on nominal *p*-values.

Pairwise correlations among miRNAs were limited, and none remained significant after FDR correction. Likewise, no correlations with age, BMI, CA125, or HE4 survived FDR correction. Full correlation results are provided in Supplementary Tables S3 and S4, with the correlation structure visualized in Supplementary Figure S7.

### 2.3. Diagnostic Performance and Adjusted Association

miR-425-5p showed the highest diagnostic performance (AUC = 0.854; 95% CI = 0.741–0.967), followed by miR-381-3p (AUC = 0.761; 95% CI = 0.616–0.907) and miR-582-5p (AUC = 0.752; 95% CI = 0.604–0.900). For miR-381-3p, lower expression values were considered indicative of ovarian cancer, consistent with its observed downregulation in malignant tissue. The remaining miRNAs showed limited discrimination (Table 3). ROC curves are shown in Supplementary Figures S1–S3.

The exploratory three-miRNA panel (miR-425-5p, miR-381-3p, and miR-582-5p) achieved an apparent AUC of 0.858 (95% CI = 0.748–0.968). The panel did not significantly outperform miR-425-5p alone (DeLong *p* = 0.596). Internal bootstrap validation using 2000 replicates indicated some optimism in the apparent performance of the multivariable model, with an optimism-corrected AUC of 0.822. The 95% bootstrap interval for test-sample AUC was 0.769–0.864. The ROC curve for the exploratory three-miRNA panel is shown in Supplementary Figure S4. Detailed internal-validation and ROC-comparison results are provided in Supplementary Tables S5–S7.

After adjustment for age and menopausal status, miR-425-5p remained associated with ovarian cancer (OR = 7.84 per 1-SD increase; 95% CI = 2.05–29.97; *p* = 0.003). miR-381-3p was not significantly associated with ovarian cancer (OR = 0.59; 95% CI = 0.29–1.20; *p* = 0.143), while miR-582-5p showed a non-significant association (OR = 6.92; 95% CI = 0.86–55.63; *p* = 0.069) (Table 4).

Model stability was further assessed using variance inflation factors (VIFs), alternative adjustment strategies, and Firth penalized logistic regression. VIFs were approximately 1.08–1.13 for the miRNA variables and 2.30–2.59 for age and menopausal status, indicating no problematic multicollinearity. In sensitivity analyses, the association between miR-425-5p and ovarian cancer remained consistent across the unadjusted and alternatively adjusted models (ORs approximately 6.89–7.84). In Firth penalized logistic regression, miR-425-5p remained significantly associated with ovarian cancer (OR = 5.50; 95% CI = 2.02–22.07; *p* < 0.001), whereas miR-381-3p and miR-582-5p were not statistically significant. Detailed sensitivity analyses are provided in Supplementary Tables S8–S10.

### 2.4. Exploratory Survival Analyses

Only four deaths and nine progression events occurred. No miRNA was significantly associated with OS or PFS in Kaplan–Meier or Cox analyses. For PFS, non-significant trends were observed for HGSC histology, interval debulking surgery, and miR-664a-3p expression. Because of the low event counts, survival findings are hypothesis-generating only. Complete survival analyses are provided in Supplementary Tables S11–S16 and Supplementary Figures S5 and S6.

## 3. Discussion

In this exploratory tissue-based study, three miRNAs showed robust differences between ovarian cancer and benign ovarian lesions after FDR correction: miR-425-5p and miR-582-5p were upregulated, whereas miR-381-3p was downregulated. Among all candidates, miR-425-5p provided the strongest individual diagnostic signal, with an AUC of 0.854, and remained associated with ovarian cancer after adjustment for age and menopausal status. This association was also retained in sensitivity analyses and Firth penalized logistic regression. In contrast, the exploratory three-miRNA panel provided only a marginal increase in apparent AUC and did not significantly outperform miR-425-5p alone. Moreover, bootstrap validation indicated optimism in the apparent performance of the multivariable panel. These findings support further independent evaluation of miR-425-5p while underscoring that the present results are hypothesis-generating rather than evidence of immediate clinical utility.

These findings are relevant because the study did not rely on a single uncorrected comparison. The three miRNAs distinguishing malignant from benign tissue remained significant after FDR correction, while several nominal subgroup and correlation findings did not. This pattern narrows the interpretation to the most reproducible signals within the dataset and reduces the risk of emphasizing chance associations generated by multiple testing. It also supports treating miR-425-5p as the leading candidate on the basis of convergent evidence—differential expression, ROC performance, and adjusted logistic regression—rather than on AUC alone.

The miR-425-5p finding is biologically plausible. Experimental studies have linked miR-425-5p to PI3K/AKT-mediated proliferation [9]. In ovarian cancer, miR-425 has also been reported to regulate proliferation, metastasis, and apoptosis through repression of PAK4 [14], a kinase involved in cytoskeletal organization, cell survival, migration, adhesion, and invasion. Increased miR-425-5p has additionally been associated with expression of cell-cycle regulators including cyclins and cyclin-dependent kinases [9]. Although these mechanistic observations were obtained in experimental systems and cannot establish causality in our cohort, they provide a coherent biological context for the higher tissue expression observed in malignant lesions. It should be noted, however, that the available evidence regarding miR-425 in ovarian cancer is not entirely consistent. In the study cited above [14], miR-425 expression was reported to be downregulated in ovarian cancer, and miR-425 suppressed cell proliferation, migration, and invasion while promoting apoptosis. Thus, the increased miR-425-5p expression observed in the present study does not fully correspond to these previously reported findings. This discrepancy may reflect differences in the biological context, cellular models, molecular targets, or study design, and the functional significance of miR-425-5p in ovarian cancer therefore remains to be fully elucidated.

miR-381-3p showed the opposite expression pattern, with lower levels in ovarian cancer. When the ROC direction was defined according to the observed expression pattern, miR-381-3p showed moderate discrimination between malignant and benign ovarian tissue (AUC = 0.761). miR-381 has been described as a tumor-suppressive regulator capable of limiting proliferation, migration, and invasion and promoting apoptosis in several malignancies [15]. Evidence in ovarian cancer also implicates miR-381 in suppression of oncogenic signaling, including regulation of YY1-related pathways [16]. In our data, the nominal inverse correlation between miR-381-3p and HE4 did not survive FDR correction, so this secondary observation should not be overinterpreted.

miR-582-5p was increased in ovarian cancer tissue and showed moderate diagnostic discrimination (AUC = 0.752). The miR-582 family appears to have context-dependent functions across malignancies. miR-582-5p has been shown to influence tumor growth and invasion through NOTCH1 signaling [10], whereas miR-582-3p has been linked to ovarian cancer progression through AKT/mTOR-related regulation [17]. The direction of effect can differ between tumor types, emphasizing that tissue context, strand specificity, and target availability are important when interpreting individual miRNAs. Our results add evidence that miR-582-5p may be relevant to ovarian cancer biology, but its independent association with cancer status did not reach conventional significance after adjustment.

The exploratory three-miRNA panel achieved an apparent AUC of 0.858, only marginally higher than that of miR-425-5p alone (AUC = 0.854), and the difference was not statistically significant (DeLong *p* = 0.596). Importantly, bootstrap internal validation demonstrated some optimism in the multivariable model, with an optimism-corrected AUC of 0.822. Thus, the present data provide no evidence that combining miR-425-5p, miR-381-3p, and miR-582-5p improves discrimination over miR-425-5p alone. Given the small cohort and the absence of an independent validation set, the panel should be regarded as exploratory. This is clinically and methodologically relevant because multi-marker panels may appear advantageous in small development cohorts while providing little reproducible incremental information. Future studies should use larger cohorts, prespecified modeling strategies, and appropriate internal and external validation; penalized approaches may also be useful when the number of candidate biomarkers increases.

Most associations with clinicopathological characteristics and most correlation analyses were null after multiplicity correction. The remaining FDR-significant association of miR-574-5p with menopausal status raises the possibility that hormonal or age-related factors influence its expression, but this finding requires replication. More broadly, miRNAs frequently regulate partly distinct networks despite overlap among their predicted targets [18], which may explain the limited pairwise correlations in our cohort. The absence of robust correlations with CA125 and HE4 could indicate that the studied miRNAs reflect biological processes not fully captured by conventional markers, but it could equally reflect limited statistical power. Circulating miRNAs have previously been investigated as complementary ovarian-cancer biomarkers [19], and combinations with established markers such as CA125 may improve performance in some settings [20]. Because our study used tumor tissue and CA125/HE4 were unavailable in controls, such combined diagnostic modeling could not be tested here.

The survival analyses did not demonstrate significant prognostic associations. Only four deaths and nine progression events were observed, producing wide confidence intervals and making stable multivariable prognostic inference impossible. The survival results should therefore be viewed as descriptive and hypothesis-generating; the lack of statistical significance cannot be interpreted as evidence that these miRNAs have no prognostic relevance. Moving the detailed Kaplan–Meier and Cox analyses to the Supplementary Material preserves transparency while keeping the main manuscript focused on the better-supported diagnostic findings.

An important strength of the analysis is the explicit treatment of multiple comparisons. Several nominal associations were observed in subgroup and correlation analyses, but most disappeared after FDR adjustment. By emphasizing the FDR-stable cancer-control differences rather than isolated nominal *p*-values, the interpretation is centered on the most reproducible signals within this dataset. This is particularly important in candidate-miRNA studies, where multiple correlated molecular measurements can otherwise generate apparently positive findings by chance. The persistence of the miR-425-5p, miR-381-3p, and miR-582-5p differences after correction therefore provides stronger support for prioritizing these candidates, while the remaining secondary findings should be considered exploratory.

The adjusted analysis also deserves cautious interpretation. Age and menopausal status differed between the malignant and benign groups and are plausible determinants of molecular expression. miR-425-5p remained significantly associated with ovarian cancer after both variables were included in the model, suggesting that the observed difference was not explained solely by these measured confounders. Nevertheless, the wide confidence interval around the adjusted odds ratio reflects the small sample and limits precision. Moreover, age and menopausal status are closely related, and other unmeasured factors may differ between women with malignant and benign ovarian disease. The adjusted result therefore strengthens the signal but does not establish independence in a causal sense.

From a translational perspective, the tissue origin of the present measurements is fundamental. A tissue biomarker may help characterize tumor biology or support future molecular classification, but it cannot by itself serve as a non-invasive screening marker because tissue is obtained during surgery or biopsy. The next clinically relevant step would be to determine whether the same expression pattern can be detected reproducibly in plasma, serum, or extracellular vesicles and whether it adds information beyond established clinical algorithms. Previous work on serum exosomal miRNAs illustrates the feasibility of this direction [19], while studies combining molecular markers with CA125 suggest that multimodal strategies may be more useful than isolated markers [20]. Any such extension should use prospectively defined thresholds and independent validation cohorts.

A clinically useful ovarian-cancer biomarker would ultimately need to add information beyond established diagnostic pathways, particularly in women presenting with an indeterminate adnexal mass. The present dataset cannot test incremental value over CA125 or HE4 because these markers were not available in the benign control group. Consequently, the observed AUC for miR-425-5p should be interpreted as discrimination between the study groups rather than proof of superiority to existing biomarkers. A logical next step would be prospective evaluation in a clinically representative cohort with paired tissue and plasma or serum samples, complete CA125 and HE4 measurements, and prespecified comparison of single-marker and combined models [3,4,20].

The targeted design has both advantages and disadvantages. Restricting the analysis to biologically selected miRNAs reduced the dimensionality of the experiment and allowed focused testing of candidates supported by the prior literature [9,10,11,12,13]. At the same time, it precluded discovery of unanticipated miRNAs that might have stronger diagnostic performance. This distinction is relevant when positioning the study: the results are best viewed as candidate validation and prioritization rather than comprehensive miRNA profiling. A future two-stage strategy, combining high-throughput discovery in one cohort with prespecified qRT-PCR validation in another, would provide a stronger framework for developing an ovarian-cancer miRNA signature.

Several limitations should be considered. First, the cohort was small, single-center, and histologically heterogeneous, which limited statistical power and may have affected the robustness and generalizability of the findings. Furthermore, the lack of external validation in an independent cohort limits the ability to assess the reproducibility and broader applicability of the proposed biomarkers. Therefore, these findings should be considered preliminary and require validation in larger, independent, preferably multicenter cohorts before their potential clinical utility can be established. Second, patients with ovarian cancer were older and more frequently postmenopausal than controls. Although adjusted logistic models were used, residual confounding cannot be excluded. Third, the candidate miRNAs were selected from prior biological evidence rather than from an unbiased genome-wide discovery platform, so potentially relevant signals may have been missed. Fourth, the three-miRNA model was developed and assessed in the same cohort and is vulnerable to overfitting. Fifth, expression was measured in tumor tissue; the findings cannot be extrapolated directly to circulating biomarkers or screening applications. CA125 and HE4 were not available in the benign control group, preventing a direct comparison or integrated model with established serum biomarkers. The relatively small sample size remains an important limitation of the multivariable analyses. Although VIF assessment did not indicate problematic multicollinearity and the miR-425-5p association remained consistent in alternative adjustment models and Firth penalized logistic regression, the confidence intervals remained wide and the estimates should be considered exploratory. These internal sensitivity analyses improve assessment of model robustness but do not substitute for external validation in an independent cohort. The clinical relevance of miR-425-5p should be interpreted cautiously. Despite an AUC of 0.854, its sensitivity was limited to 62.5% at the Youden-optimal threshold, with a specificity of 95.5%. Furthermore, the exploratory three-miRNA panel did not significantly outperform miR-425-5p alone (DeLong *p* = 0.596). As no direct comparison with established serum biomarkers was possible, incremental clinical diagnostic value remains unproven. An additional important limitation is that miRNA expression was assessed in bulk tissue specimens without quantitative evaluation of tumor cellularity or microdissection. Consequently, histological heterogeneity and differences in the cellular composition of the analyzed tissue specimens, including varying proportions of tumor cells, stromal cells, and other non-tumor cell populations, may have contributed to the observed variability in miRNA expression. These factors should be taken into account when interpreting the results, as differences in tissue composition may affect the measured miRNA expression profiles independently of tumor-specific biological differences. However, all tissue samples included in the miRNA analysis were collected before the initiation of neoadjuvant chemotherapy; thus, the potential confounding effect of treatment-related changes in miRNA expression is unlikely to have affected the present analysis. Future work should combine discovery approaches such as small-RNA sequencing with targeted validation in larger, independent and clinically well-characterized cohorts, followed by functional studies of the most reproducible candidates.

## 4. Materials and Methods

### 4.1. Study Design and Patients

The study included 46 women undergoing surgery for an ovarian mass: 24 with histopathologically confirmed epithelial ovarian cancer and 22 with benign ovarian lesions. Tumor tissue was collected during surgery for molecular analysis. Exclusion criteria were another current or previous primary malignancy, acute inflammatory disease, active infection, exacerbation of chronic disease, or incomplete clinicopathological data. FIGO stage, histological type, age, body mass index (BMI), menopausal status, and pre-treatment CA125 and HE4 were recorded. Histology was grouped as high-grade serous carcinoma (HGSC) or other epithelial subtypes (clear cell, endometrioid, low-grade serous, and mucinous). Reference values were CA125 < 35 U/mL and HE4 < 70 pmol/L; threshold-based subgroup analyses were not performed because few cancer patients had values below these limits and measurements were unavailable in controls. Of the 24 patients with ovarian cancer, 20 underwent primary debulking surgery (PDS), 3 were assigned to neoadjuvant chemotherapy (NACT) followed by interval debulking surgery (IDS), and 1 died before treatment was initiated. In the PDS group, tissue was collected during primary surgery, whereas in patients assigned to NACT, tumor tissue was obtained during diagnostic laparoscopy before the initiation of chemotherapy. Thus, all tumor samples analyzed for miRNA expression were treatment-naïve. Tumor tissue specimens were obtained directly from the tumor during primary surgery or diagnostic laparoscopy, as appropriate. The study was approved by the Bioethics Committee of the Pomeranian Medical University in Szczecin (KB-0012/55/2021; 20 December 2021), and all participants provided written informed consent.

### 4.2. RNA Isolation and miRNA Quantification

Total RNA was isolated from 25 mg of tissue using the Direct-zol RNA MiniPrep kit (Zymo Research, Irvine, CA, USA). RNA concentration was measured by NanoDrop spectrophotometry and purity was accepted at OD260/280 ≥ 1.8. cDNA was synthesized from 5 ng total RNA with the TaqMan Advanced miRNA cDNA Synthesis Kit (Thermo Fisher Scientific, Waltham, MA, USA). TaqMan Advanced miRNA Assays were used for hsa-miR-664a-3p (478193), miR-432-3p (478892), miR-381-3p (477816), miR-582-5p (478166), miR-499a-3p (478948), miR-612 (479095), miR-574-5p (479357), miR-889-3p (478208), miR-505-5p (478957), and miR-425-5p (478094). qRT-PCR was performed on a QuantStudio 7 Pro system in two technical replicates (10 µL reactions). Assay thresholds were set manually and mean cycle-threshold (Ct) values were calculated for replicates.

### 4.3. Statistical Analysis

Five candidate reference miRNAs (hsa-miR-103a-3p, hsa-miR-191-5p, hsa-miR-361-5p, let-7e-5p, and miR-423-3p) were initially quantified in all samples. Reference-miRNA stability was evaluated jointly across all malignant and benign samples using the endogenous control assessment. Because the analyzed dataset comprised heterogeneous tissue samples, an endogenous control score < 1.0, in accordance with the manufacturer’s recommendation, was used as the stability criterion. Three miRNAs—hsa-miR-103a-3p, hsa-miR-191-5p, and hsa-miR-361-5p—met this criterion and were selected as reference miRNAs, whereas the remaining candidates had scores > 1.0. The arithmetic mean of the Ct values of the three selected reference miRNAs was used as the reference Ct, corresponding to the geometric mean of their expression quantities under the assumption of comparable amplification efficiencies. ΔCt was calculated as Ct(target miRNA)—Ct(reference), and relative expression was calculated as 2^−ΔCt. Target miRNAs with Ct ≥ 35 were considered below the detection threshold. Four candidate miRNAs (miR-432-3p, miR-499a-3p, miR-612, and miR-889-3p) were excluded from further analyses because they were below the detection threshold in the majority or all samples. The proportions of samples with undetectable expression of these miRNAs did not differ significantly between the malignant and benign groups (Fisher’s exact test). The final analysis therefore included miR-664a-3p, miR-381-3p, miR-582-5p, miR-574-5p, miR-505-5p, and miR-425-5p.

Data distribution was assessed using the Shapiro–Wilk test. Non-normally distributed continuous variables are reported as median and interquartile range and were compared using the Mann–Whitney U test; categorical variables were compared using the chi-square or Fisher exact test. Spearman coefficients were used for correlations. Prespecified comparisons included cancer versus benign lesions, FIGO I-II versus III-IV, HGSC versus other histologies, BMI ≤ 25 versus >25 kg/m^2^, and menopausal status.

Diagnostic performance was evaluated using receiver operating characteristic (ROC) analysis performed in RStudio version 2026.09.0 using the pROC package. The area under the curve (AUC), sensitivity, specificity, and optimal cut-off based on the Youden index were calculated. ROC direction was defined according to the observed direction of differential expression. Correlated ROC curves were compared using the non-parametric DeLong test implemented in the pROC package.

Internal validation of individual-miRNA diagnostic performance was performed using bootstrap resampling with 2000 replicates. For the exploratory three-miRNA logistic model, bootstrap validation additionally quantified model optimism by comparing model performance in bootstrap training samples with performance on the original dataset. An optimism-corrected AUC was subsequently calculated.

Potential multicollinearity in the adjusted logistic regression models was assessed using variance inflation factors (VIFs). Model robustness was further examined using sensitivity analyses with sequential adjustment for age and menopausal status. Because of the limited sample size and the potential for small-sample bias in maximum-likelihood logistic regression, Firth penalized logistic regression was additionally performed as a sensitivity analysis. Odds ratios for miRNA expression were expressed per one-standard-deviation increase.

For survival analyses, patients were dichotomized at the median miRNA expression; PFS and OS were evaluated using Kaplan–Meier curves and log-rank tests, with univariate and multivariable Cox proportional hazards models. Benjamini–Hochberg false discovery rate (FDR) correction was applied separately within prespecified families of cancer-control/subgroup comparisons and correlation analyses. Two-sided *p* < 0.05 was considered statistically significant, with results interpreted as hypothesis-generating given the exploratory design.

Statistical analyses were performed using StatView version 5.0 (SAS Institute, Cary, NC, USA), Statistica version 13.3 (TIBCO Software Inc., Palo Alto, CA, USA), R version 4.3.2 (R Foundation for Statistical Computing, Vienna, Austria), RStudio version 2026.09.0, and GraphPad Prism 7 (GraphPad Software, San Diego, CA, USA).

## 5. Conclusions

miR-425-5p, miR-381-3p, and miR-582-5p were differentially expressed between ovarian cancer and benign ovarian lesions after FDR correction. miR-425-5p showed the highest individual diagnostic accuracy and remained associated with ovarian cancer after adjustment for age and menopausal status, with consistent results in sensitivity and Firth penalized regression analyses. The exploratory three-miRNA panel did not significantly improve discrimination and showed some optimism on bootstrap internal validation. These tissue-based findings support miR-425-5p as a candidate for further independent investigation but require confirmation in larger external cohorts before any clinical application can be considered. Overall, these findings should be interpreted as exploratory and hypothesis-generating rather than as evidence of established clinical utility.

## Figures and Tables

**Table 1:** Clinicodemographic characteristics of the study population.

Clinicodemographic Characteristics	Total Cohort (*n* = 46)	Ovarian Cancer (*n* = 24)	Benign Ovarian Lesions (*n* = 22)	*p*-Value
**Continuous variables, median (IQR)**				
Age (years)	58.6 (47.2–70.4)	67.1 (54.1–70.1)	48.3 (34.5–69.4)	0.02
BMI (kg/m^2^)	25.9 (23.0–29.1)	26.1 (23.9–31.2)	24.8 (22.4–27.4)	0.16
**Categorical variables, n (%)**				
**Age (years)**				**0.06**
<65	27 (58.7%)	11 (45.8%)	16 (72.7%)	
≥65	19 (41.3%)	13 (54.2%)	6 (27.3%)	
**BMI (kg/m^2^)**				**0.25**
≤25	21 (45.7%)	9 (37.5%)	12 (54.5%)	
>25	25 (54.3%)	15 (62.5%)	10 (45.5%)	
**Menopausal status**				**0.04**
Premenopausal	18 (39.1%)	6 (25.0%)	12 (54.5%)	
Postmenopausal	28 (60.9%)	18 (75.0%)	10 (45.5%)	
**Clinicopathological characteristics of ovarian cancer patients**				
Variable		*n* (%)		
FIGO I–II		8 (33.3%)		
FIGO III–IV		16 (66.7%)		
High-grade serous carcinoma		16 (66.7%)		
Other histological types *		8 (33.3%)		

Data are presented as median (interquartile range) or *n* (%). Continuous variables were compared using the Mann–Whitney U test; categorical variables were compared using the chi-square test or Fisher’s exact test, as appropriate. BMI, body mass index; IQR, interquartile range. * Other histological types included clear cell (*n* = 2), endometrioid (*n* = 1), low-grade serous (*n* = 2), and mucinous (*n* = 3) ovarian carcinomas.

**Table 2:** Expression of miRNAs with significant cancer-control differences.

miRNA	Ovarian Cancer, Median (IQR)	Benign Lesions, Median (IQR)	*p*-Value
miR-381-3p	0.01 (0.00–0.05)	0.06 (0.03–0.13)	0.002
miR-582-5p	0.02 (0.01–0.03)	0.00 (0.00–0.01)	0.003
miR-425-5p	0.43 (0.23–0.65)	0.14 (0.09–0.21)	<0.001

Mann–Whitney U tests. All three associations remained significant after Benjamini–Hochberg FDR correction (q = 0.030).

**Table 3:** ROC analysis of selected miRNAs for discrimination of ovarian cancer.

miRNA	AUC (95% CI)	Sensitivity (TPR)	Specificity (TNR)	Youden Index	Optimal Cut-Off
miR-425-5p	0.854 (0.741–0.967)	0.625	0.955	0.580	0.393
miR-381-3p	0.761 (0.616–0.907)	0.708	0.864	0.572	0.01066
miR-582-5p	0.752 (0.604–0.900)	0.917	0.545	0.462	0.001862
miR-574-5p	0.667 (0.507–0.827)	1.000	0.318	0.318	0.419492
miR-505-5p	0.529 (0.357–0.701)	0.625	0.545	0.170	0.002453
miR-664a-3p	0.500 (0.329–0.671)	0.333	0.773	0.106	0.059887

AUC, area under the curve; CI, confidence interval; TPR, true-positive rate; TNR, true-negative rate. ROC direction was defined according to the observed direction of differential expression. For miR-381-3p, miR-574-5p, and miR-664a-3p, lower expression values were considered indicative of ovarian cancer; for miR-425-5p, miR-582-5p, and miR-505-5p, higher expression values were considered indicative of ovarian cancer. Optimal cut-offs were determined using the Youden index.

**Table 4:** Multivariable logistic regression adjusted for age and menopausal status.

Biomarker	Adjusted OR	95% CI	*p*-Value
miR-425-5p	7.84	2.05–29.97	0.003
miR-381-3p	0.59	0.29–1.20	0.143
miR-582-5p	6.92	0.86–55.63	0.069

ORs are from separate models and represent a one-standard-deviation increase in miRNA expression. Each model was adjusted for age and menopausal status.

## Data Availability

The original contributions presented in this study are included in the article/Supplementary Material. Further inquiries can be directed to the corresponding author.

## Supplementary Materials

The following supporting information can be downloaded at: https://www.mdpi.com/article/10.3390/ijms27209122/s1.
