# Supplementary material for: Exploratory Analysis of Selected MicroRNAs as Tissue-Based Candidate Biomarkers in Ovarian Cancer
Source: Int J Mol Sci. 2026 Oct 15;27(20):9122. doi: 10.3390/ijms27209122 (PMC13705106; doi:10.3390/ijms27209122)

Supplementary

Table S1. Associations remaining significant after FDR correction

Comparison	miRNA	Raw p-value	FDR-adjusted p-value
Ovarian cancer vs benign ovarian lesions	miR-425-5p	<0.001	0.030
Ovarian cancer vs benign ovarian lesions	miR-381-3p	0.002	0.030
Ovarian cancer vs benign ovarian lesions	miR-582-5p	0.003	0.030
Premenopausal vs postmenopausal	miR-574-5p	0.004	0.030

Benjamini-Hochberg FDR correction was applied to comparisons in the full clinicopathological analysis. Only significant adjusted associations are shown.

Table S2. Complete comparison of miRNA expression levels according to clinicopathological parameters

Parameter	miR-664a-3p Mdn (IQR)	p - value	miR-381-3p Mdn (IQR)	p - value	miR-582-5p Mdn (IQR)	p - value	miR-574-5p Mdn (IQR)	p - value	miR-505-5p Mdn (IQR)	p - value	miR-425-5p Mdn (IQR)	p - value
FIGO I–II (n=8)	0.12 (0.09–0.24)	0.07	0.01 (0.00–0.09)	0.13	0.02 (0.00–0.02)	0.44	0.12 (0.05–0.19)	0.88	0.00 (0.00–0.01)	0.83	0.36 (0.17–0.48)	0.17
FIGO III–IV (n=16)	0.06 (0.05–0.15)	0.07	0.00 (0.00–0.02)	0.13	0.02 (0.01–0.04)	0.44	0.08 (0.06–0.21)	0.88	0.00 (0.00–0.01)	0.83	0.48 (0.25–0.80)	0.17
Ovarian cancer (n=24)	0.08 (0.06–0.20)	1.00	0.01 (0.00–0.05)	0.002	0.02 (0.01–0.03)	0.003	0.09 (0.06–0.19)	0.05	0.00 (0.00–0.01)	0.74	0.43 (0.23–0.65)	<0.001
Benign ovarian lesions (n=22)	0.09 (0.06–0.16)	1.00	0.06 (0.03–0.13)	0.002	0.00 (0.00–0.01)	0.003	0.16 (0.08–0.46)	0.05	0.00 (0.00–0.00)	0.74	0.14 (0.09–0.21)	<0.001
BMI ≤25 (n=21)	0.09 (0.06–0.21)	0.50	0.03 (0.01–0.11)	0.45	0.01 (0.00–0.02)	0.98	0.13 (0.05–0.34)	0.76	0.00 (0.00–0.00)	0.77	0.26 (0.12–0.45)	0.81
BMI >25 (n=25)	0.08 (0.06–0.15)	0.50	0.01 (0.00–0.11)	0.45	0.01 (0.00–0.03)	0.98	0.14 (0.07–0.27)	0.76	0.00 (0.00–0.01)	0.77	0.21 (0.13–0.44)	0.81
Premenopausal (n=18)	0.14 (0.06–0.22)	0.03	0.04 (0.01–0.12)	0.42	0.01 (0.00–0.04)	0.74	0.26 (0.12–0.46)	0.004	0.00 (0.00–0.01)	0.29	0.19 (0.12–0.39)	0.81
Postmenopausal (n=28)	0.08 (0.05–0.11)	0.03	0.01 (0.00–0.11)	0.42	0.01 (0.00–0.02)	0.74	0.08 (0.05–0.18)	0.004	0.00 (0.00–0.01)	0.29	0.24 (0.12–0.48)	0.81
High-grade serous (n=16)	0.07 (0.05–0.16)	0.12	0.00 (0.00–0.01)	0.18	0.02 (0.01–0.03)	0.88	0.08 (0.06–0.15)	0.44	0.00 (0.00–0.00)	0.03	0.48 (0.32–0.76)	0.13
Other subtypes (n=8)	0.12 (0.08–0.26)	0.12	0.02 (0.00–0.09)	0.18	0.01 (0.01–0.04)	0.88	0.16 (0.06–0.24)	0.44	0.01 (0.00–0.01)	0.03	0.28 (0.17–0.45)	0.13

Data are median (IQR). Mann-Whitney U tests were used. Histological comparison: HGSC versus other epithelial subtypes.

Table S3. Pairwise correlations between analyzed miRNAs in ovarian cancer and control groups

miRNA pair	Control group (n=22)	Control group (n=22)	Ovarian cancer group (n=24)	Ovarian cancer group (n=24)
miRNA pair	ρ	p-value	ρ	p-value
miR-664a-3p vs miR-381-3p	—	—	0.24	0.26
miR-664a-3p vs miR-582-5p	—	—	0.22	0.31
miR-664a-3p vs miR-574-5p	—	—	0.49	0.02*
miR-664a-3p vs miR-505-5p	—	—	0.29	0.16
miR-664a-3p vs miR-425-5p	—	—	-0.20	0.34
miR-381-3p vs miR-582-5p	—	—	-0.24	0.27
miR-381-3p vs miR-574-5p	—	—	0.11	0.59
miR-381-3p vs miR-505-5p	—	—	0.04	0.86
miR-381-3p vs miR-425-5p	—	—	-0.51	0.01*
miR-582-5p vs miR-574-5p	—	—	0.19	0.36
miR-582-5p vs miR-505-5p	—	—	0.19	0.35
miR-582-5p vs miR-425-5p	—	—	0.38	0.07
miR-574-5p vs miR-505-5p	0.52	0.01*	0.45	0.03*
miR-574-5p vs miR-425-5p	—	—	0.21	0.33
miR-505-5p vs miR-425-5p	—	—	0.27	0.21

Spearman correlation coefficients (ρ) with p-values. After FDR correction, none remained statistically significant.

Table S4. Correlation between miRNA expression and clinical/laboratory parameters in ovarian cancer patients

**miRNA vs parameter**	**Ovarian cancer group (n=24)**	**Ovarian cancer group (n=24)**
miRNA vs parameter	ρ	p-value
miR-664a-3p vs CA125 (U/mL)	-0.24	0.26
miR-664a-3p vs HE4 (pmol/L)	-0.19	0.40
miR-664a-3p vs Age	-0.30	0.16
miR-664a-3p vs BMI	-0.33	0.11
miR-381-3p vs CA125 (U/mL)	-0.40	0.06
miR-381-3p vs HE4 (pmol/L)	-0.47	0.03*
miR-381-3p vs Age	-0.20	0.35
miR-381-3p vs BMI	-0.14	0.51
miR-582-5p vs CA125 (U/mL)	-0.03	0.91
miR-582-5p vs HE4 (pmol/L)	0.11	0.62
miR-582-5p vs Age	-0.23	0.28
miR-582-5p vs BMI	-0.25	0.25
miR-574-5p vs CA125 (U/mL)	-0.40	0.06
miR-574-5p vs HE4 (pmol/L)	-0.35	0.11
miR-574-5p vs Age	-0.25	0.24
miR-574-5p vs BMI	-0.07	0.76
miR-505-5p vs CA125 (U/mL)	-0.10	0.66
miR-505-5p vs HE4 (pmol/L)	-0.19	0.39
miR-505-5p vs Age	-0.18	0.40
miR-505-5p vs BMI	-0.20	0.36
miR-425-5p vs CA125 (U/mL)	0.22	0.32
miR-425-5p vs HE4 (pmol/L)	0.22	0.32
miR-425-5p vs Age	-0.21	0.33
miR-425-5p vs BMI	-0.06	0.77

Spearman correlation coefficients (ρ) with p-values. After FDR correction, none remained statistically significant.

Table S5. Internal bootstrap validation of diagnostic performance of the three principal miRNAs

**Marker**	**Apparent AUC**	**Bootstrap AUC mean (95% bootstrap interval)**	**Bootstrap sensitivity mean (95% bootstrap interval)**	**Bootstrap specificity mean (95% bootstrap interval)**	**Bootstrap cut-off median (95% bootstrap interval)**
miR-425-5p	0.854	0.854 (0.735–0.956)	0.791 (0.542–1.000)	0.867 (0.591–1.000)	0.25902 (0.15342–0.40232)
miR-381-3p	0.761	0.760 (0.612–0.894)	0.718 (0.500–0.917)	0.858 (0.636–1.000)	0.01744 (0.00184–0.09564)
miR-582-5p	0.752	0.751 (0.593–0.891)	0.833 (0.583–1.000)	0.690 (0.409–0.955)	0.00786 (0.00070–0.01363)

Bootstrap validation was performed using 2,000 successful replicates for each marker. For miR-381-3p, lower expression levels were indicative of ovarian cancer, and the ROC direction was specified accordingly. Bootstrap AUC, sensitivity, and specificity are presented as mean values across bootstrap replicates, whereas the optimal cut-off is reported as the median with its 95% bootstrap interval. Optimal cut-offs were recalculated independently within each bootstrap sample and may therefore differ from those determined using the original full cohort and reported in Table 3 of the main manuscript.

Table S6. Bootstrap internal validation of the exploratory three-miRNA panel

**Metric**	**Value**
Apparent AUC (95% CI)	0.858 (0.748–0.968)
Bootstrap replicates	2,000
Mean bootstrap training AUC	0.869
Mean bootstrap test AUC	0.833
Mean optimism	0.036
Optimism-corrected AUC	0.822
Bootstrap test AUC 95% interval	0.769–0.864

The three-miRNA panel comprised miR-425-5p, miR-381-3p, and miR-582-5p. Optimism was estimated as the mean difference between bootstrap training performance and performance when the bootstrap-fitted model was evaluated on the original sample. The optimism-corrected AUC was calculated by subtracting mean optimism from the apparent AUC. The bootstrap test AUC interval describes the distribution of test performance and is not a confidence interval for the optimism-corrected AUC.

Table S7. Pairwise comparison of ROC curves using DeLong's test

Comparison	AUC 1	AUC 2	ΔAUC (1–2)	DeLong p-value
miR-425-5p vs miR-381-3p	0.854	0.761	0.093	0.182
miR-425-5p vs miR-582-5p	0.854	0.752	0.102	0.226
miR-381-3p vs miR-582-5p	0.761	0.752	0.009	0.912
miR-425-5p vs 3-miRNA panel	0.854	0.858	−0.004	0.596
miR-381-3p vs 3-miRNA panel	0.761	0.858	−0.097	0.168
miR-582-5p vs 3-miRNA panel	0.752	0.858	−0.106	0.199

All ROC comparisons were based on 46 paired observations. ROC direction was specified according to the observed direction of differential expression; for miR-381-3p, lower expression indicated ovarian cancer.

Table S8. Sensitivity analyses of logistic regression models for ovarian cancer status

Marker	Model	OR per 1 SD	95% CI	p-value
miR-425-5p	miRNA alone	6.89	1.90–24.99	0.003
miR-425-5p	miRNA + age	7.84	2.05–29.97	0.003
miR-425-5p	miRNA + menopausal status	7.65	2.03–28.78	0.003
miR-425-5p	miRNA + age + menopausal status	7.84	2.05–29.97	0.003
miR-381-3p	miRNA alone	0.53	0.26–1.10	0.088
miR-381-3p	miRNA + age	0.58	0.30–1.16	0.124
miR-381-3p	miRNA + menopausal status	0.51	0.25–1.02	0.057
miR-381-3p	miRNA + age + menopausal status	0.59	0.29–1.20	0.143
miR-582-5p	miRNA alone	4.69	0.78–28.32	0.092
miR-582-5p	miRNA + age	7.12	0.91–55.55	0.061
miR-582-5p	miRNA + menopausal status	7.28	1.01–52.38	0.049
miR-582-5p	miRNA + age + menopausal status	6.92	0.86–55.63	0.069

Odds ratios represent a one-standard-deviation increase in miRNA expression. Each model included n=46 observations and converged successfully.

Table S9. Variance inflation factors for the fully adjusted logistic regression models

Marker model	Variable	VIF
miR-425-5p	miRNA	1.13
miR-425-5p	Age	2.42
miR-425-5p	Menopausal status	2.30
miR-381-3p	miRNA	1.08
miR-381-3p	Age	2.50
miR-381-3p	Menopausal status	2.59
miR-582-5p	miRNA	1.13
miR-582-5p	Age	2.42
miR-582-5p	Menopausal status	2.46

VIF, variance inflation factor. Values were calculated for models including the indicated miRNA, age, and menopausal status (n=46).

Table S10. Firth penalized logistic regression sensitivity analysis

Marker	OR per 1 SD	95% CI	p-value
miR-425-5p	5.50	2.02–22.07	<0.001
miR-381-3p	0.63	0.30–1.16	0.141
miR-582-5p	5.04	0.85–42.92	0.129

Firth penalized logistic regression was used as a small-sample sensitivity analysis (n=46). Odds ratios represent a one-standard-deviation increase in miRNA expression.

Table S11. Univariate Cox proportional hazards regression analysis for progression-free survival

Variable	HR	95% CI	p-value
Age (per year)	1.041	0.979–1.106	0.200
FIGO III–IV vs I–II	3.744	0.729–19.236	0.114
High-grade serous vs Other	8.291	0.961–71.493	0.054
IDS vs PDS	3.587	0.916–14.043	0.067
miR-664a-3p (per 1 SD)	0.448	0.188–1.066	0.069
miR-381-3p (per 1 SD)	1.217	0.736–2.014	0.444
miR-582-5p (per 1 SD)	0.474	0.137–1.641	0.239
miR-574-5p (per 1 SD)	0.706	0.327–1.524	0.375
miR-505-5p (per 1 SD)	0.689	0.311–1.526	0.358
miR-425-5p (per 1 SD)	1.226	0.664–2.261	0.515

Table S12. Kaplan-Meier analysis of overall survival (OS)

Endpoint	miRNA	Cutoff (median)	n High	Events High	Median surv. High (mo)	n Low	Events Low	Median surv. Low (mo)	Log-rank chi²	p-value
OS	miR-664a-3p	0.081466	12	2	NR	12	2	NR	0.004	0.9472
OS	miR-381-3p	0.005230	12	2	NR	12	2	NR	0.002	0.9658
OS	miR-582-5p	0.015702	12	1	NR	12	3	NR	1.866	0.1720
OS	miR-574-5p	0.089221	12	1	NR	12	3	NR	1.050	0.3054
OS	miR-505-5p	0.002907	12	2	NR	12	2	NR	0.001	0.9819
OS	miR-425-5p	0.427198	12	2	NR	12	2	NR	0.001	0.9749

Table S13. Univariate Cox regression analysis for overall survival (OS)

Endpoint	Variable	n	Events	HR	95% CI lower	95% CI upper	p-value
OS	Age (per year)	24	4	1.050	0.959	1.149	0.2930
OS	FIGO III-IV (vs I-II)	24	4	4.808	0.436	52.978	0.1996
OS	HGSC (vs Other)	24	4	4.233	0.365	49.105	0.2486
OS	IDS (vs PDS)	24	4	5.669	0.888	36.205	0.0666
OS	miR-664a-3p (per 1 SD)	24	4	0.707	0.261	1.912	0.4940
OS	miR-664a-3p High vs Low	24	4	0.951	0.172	5.271	0.9540
OS	miR-381-3p (per 1 SD)	24	4	1.118	0.524	2.388	0.7731
OS	miR-381-3p High vs Low	24	4	1.033	0.186	5.747	0.9703
OS	miR-582-5p (per 1 SD)	24	4	0.620	0.155	2.475	0.4981
OS	miR-582-5p High vs Low	24	4	0.350	0.058	2.103	0.2510
OS	miR-574-5p (per 1 SD)	24	4	1.064	0.442	2.560	0.8906
OS	miR-574-5p High vs Low	24	4	0.445	0.073	2.722	0.3807
OS	miR-505-5p (per 1 SD)	24	4	0.951	0.352	2.573	0.9214
OS	miR-505-5p High vs Low	24	4	0.983	0.177	5.452	0.9842
OS	miR-425-5p (per 1 SD)	24	4	1.238	0.544	2.814	0.6109
OS	miR-425-5p High vs Low	24	4	0.976	0.176	5.406	0.9781

Table S14. Multivariable Cox proportional hazards regression analysis for overall survival (OS)

Endpoint	miRNA model	Variable	n	Events	HR	95% CI lower	95% CI upper	p-value
OS	miR-664a-3p	Age	24	4	1.013	0.964	1.064	0.6110
OS	miR-664a-3p	FIGO III-IV	24	4	1.411	0.491	4.061	0.5227
OS	miR-664a-3p	HGSC	24	4	1.325	0.454	3.864	0.6061
OS	miR-664a-3p	IDS vs PDS	24	4	1.909	0.491	7.424	0.3510
OS	miR-664a-3p	miR-664a-3p (per 1 SD)	24	4	0.957	0.577	1.586	0.8636
OS	miR-381-3p	Age	24	4	1.013	0.965	1.064	0.5962
OS	miR-381-3p	FIGO III-IV	24	4	1.414	0.492	4.064	0.5203
OS	miR-381-3p	HGSC	24	4	1.331	0.458	3.872	0.5994
OS	miR-381-3p	IDS vs PDS	24	4	1.963	0.505	7.636	0.3305
OS	miR-381-3p	miR-381-3p (per 1 SD)	24	4	1.058	0.654	1.711	0.8179
OS	miR-582-5p	Age	24	4	1.013	0.965	1.064	0.5995
OS	miR-582-5p	FIGO III-IV	24	4	1.417	0.494	4.069	0.5172
OS	miR-582-5p	HGSC	24	4	1.327	0.456	3.862	0.6038
OS	miR-582-5p	IDS vs PDS	24	4	1.929	0.500	7.444	0.3403
OS	miR-582-5p	miR-582-5p (per 1 SD)	24	4	0.915	0.540	1.553	0.7430
OS	miR-574-5p	Age	24	4	1.013	0.965	1.064	0.5959
OS	miR-574-5p	FIGO III-IV	24	4	1.415	0.492	4.066	0.5193
OS	miR-574-5p	HGSC	24	4	1.331	0.458	3.872	0.5997
OS	miR-574-5p	IDS vs PDS	24	4	1.963	0.503	7.663	0.3319
OS	miR-574-5p	miR-574-5p (per 1 SD)	24	4	1.044	0.633	1.722	0.8649
OS	miR-505-5p	Age	24	4	1.013	0.965	1.064	0.6004
OS	miR-505-5p	FIGO III-IV	24	4	1.417	0.493	4.070	0.5171
OS	miR-505-5p	HGSC	24	4	1.333	0.457	3.883	0.5989
OS	miR-505-5p	IDS vs PDS	24	4	1.937	0.502	7.483	0.3374
OS	miR-505-5p	miR-505-5p (per 1 SD)	24	4	1.005	0.598	1.689	0.9843
OS	miR-425-5p	Age	24	4	1.013	0.965	1.064	0.5997
OS	miR-425-5p	FIGO III-IV	24	4	1.413	0.491	4.068	0.5218
OS	miR-425-5p	HGSC	24	4	1.326	0.454	3.874	0.6054
OS	miR-425-5p	IDS vs PDS	24	4	1.926	0.496	7.471	0.3435
OS	miR-425-5p	miR-425-5p (per 1 SD)	24	4	1.023	0.634	1.649	0.9263

Table S15. Multivariable Cox proportional hazards regression analysis for progression-free survival (PFS)

Endpoint	miRNA model	Variable	n	Events	HR	95% CI lower	95% CI upper	p-value
PFS	miR-664a-3p	Age	24	9	1.028	0.968	1.090	0.3702
PFS	miR-664a-3p	FIGO III-IV	24	9	1.847	0.495	6.896	0.3613
PFS	miR-664a-3p	HGSC	24	9	2.619	0.635	10.806	0.1829
PFS	miR-664a-3p	IDS vs PDS	24	9	1.268	0.317	5.083	0.7371
PFS	miR-664a-3p	miR-664a-3p (per 1 SD)	24	9	0.746	0.393	1.415	0.3699
PFS	miR-381-3p	Age	24	9	1.036	0.976	1.099	0.2481
PFS	miR-381-3p	FIGO III-IV	24	9	1.886	0.501	7.101	0.3483
PFS	miR-381-3p	HGSC	24	9	2.740	0.670	11.208	0.1607
PFS	miR-381-3p	IDS vs PDS	24	9	1.682	0.413	6.843	0.4677
PFS	miR-381-3p	miR-381-3p (per 1 SD)	24	9	1.209	0.768	1.904	0.4123
PFS	miR-582-5p	Age	24	9	1.036	0.977	1.099	0.2394
PFS	miR-582-5p	FIGO III-IV	24	9	2.154	0.584	7.942	0.2493
PFS	miR-582-5p	HGSC	24	9	2.785	0.690	11.242	0.1502
PFS	miR-582-5p	IDS vs PDS	24	9	1.380	0.349	5.450	0.6459
PFS	miR-582-5p	miR-582-5p (per 1 SD)	24	9	0.650	0.304	1.390	0.2668
PFS	miR-574-5p	Age	24	9	1.032	0.973	1.094	0.2967
PFS	miR-574-5p	FIGO III-IV	24	9	2.034	0.557	7.427	0.2825
PFS	miR-574-5p	HGSC	24	9	2.767	0.689	11.111	0.1512
PFS	miR-574-5p	IDS vs PDS	24	9	1.351	0.334	5.462	0.6727
PFS	miR-574-5p	miR-574-5p (per 1 SD)	24	9	0.829	0.438	1.566	0.5630
PFS	miR-505-5p	Age	24	9	1.034	0.975	1.096	0.2702
PFS	miR-505-5p	FIGO III-IV	24	9	1.993	0.543	7.310	0.2982
PFS	miR-505-5p	HGSC	24	9	2.618	0.630	10.875	0.1853
PFS	miR-505-5p	IDS vs PDS	24	9	1.467	0.374	5.756	0.5829
PFS	miR-505-5p	miR-505-5p (per 1 SD)	24	9	0.878	0.454	1.695	0.6977
PFS	miR-425-5p	Age	24	9	1.033	0.975	1.096	0.2720
PFS	miR-425-5p	FIGO III-IV	24	9	1.970	0.534	7.274	0.3087
PFS	miR-425-5p	HGSC	24	9	2.752	0.673	11.263	0.1590
PFS	miR-425-5p	IDS vs PDS	24	9	1.514	0.386	5.933	0.5517
PFS	miR-425-5p	miR-425-5p (per 1 SD)	24	9	0.989	0.592	1.653	0.9669

Table S16. Kaplan-Meier survival analysis of progression-free survival (PFS) according to miRNA expression levels

Endpoint	miRNA	Cutoff (median)	n High	Events High	Median surv. High (mo)	n Low	Events Low	Median surv. Low (mo)	Log-rank chi²	p-value
PFS	miR-664a-3p	0.081466	12	3	NR	12	6	20.73	1.622	0.2029
PFS	miR-381-3p	0.005230	12	3	NR	12	6	23.69	0.982	0.3217
PFS	miR-582-5p	0.015702	12	4	NR	12	5	20.73	0.960	0.3271
PFS	miR-574-5p	0.089221	12	2	NR	12	7	20.73	3.109	0.0779
PFS	miR-505-5p	0.002907	12	3	NR	12	6	23.69	1.115	0.2910
PFS	miR-425-5p	0.427198	12	5	NR	12	4	NR	0.231	0.6307

Figure S1. ROC curve for miR-425-5p (AUC=0.854)

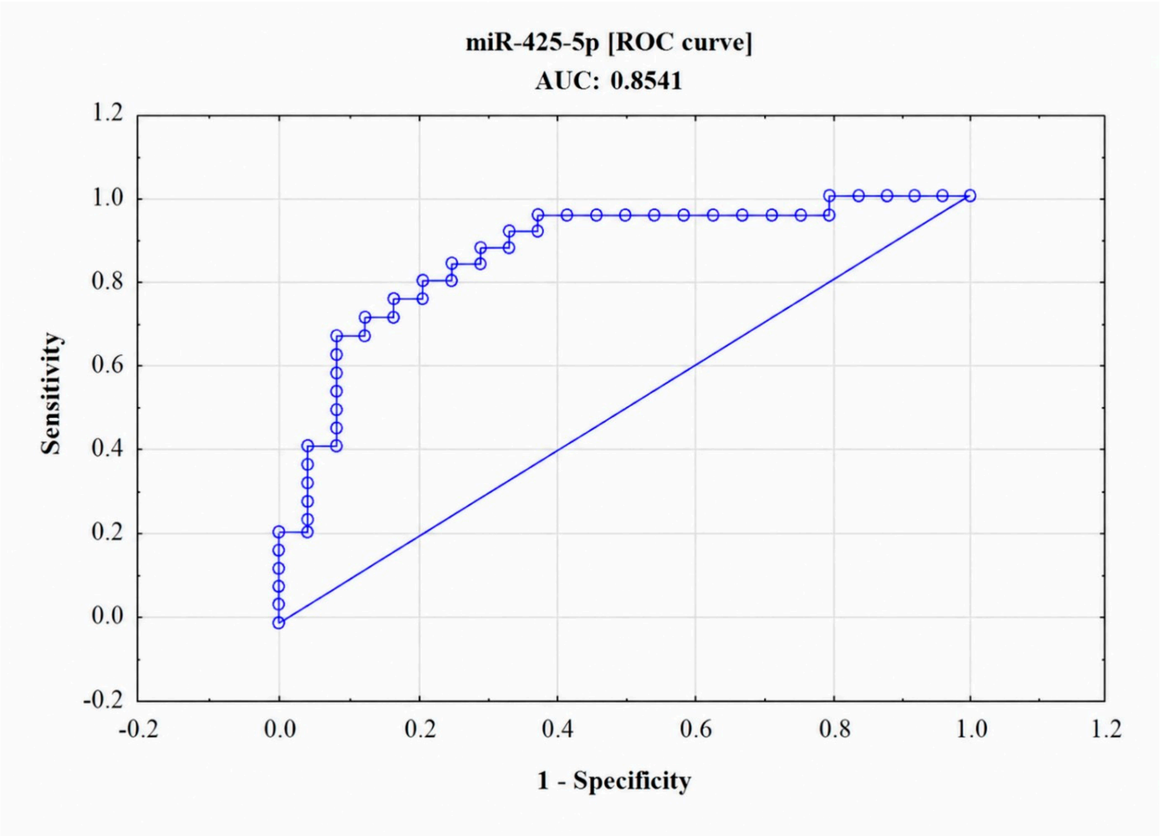

Figure S2. ROC curve for miR-582-5p (AUC=0.752)

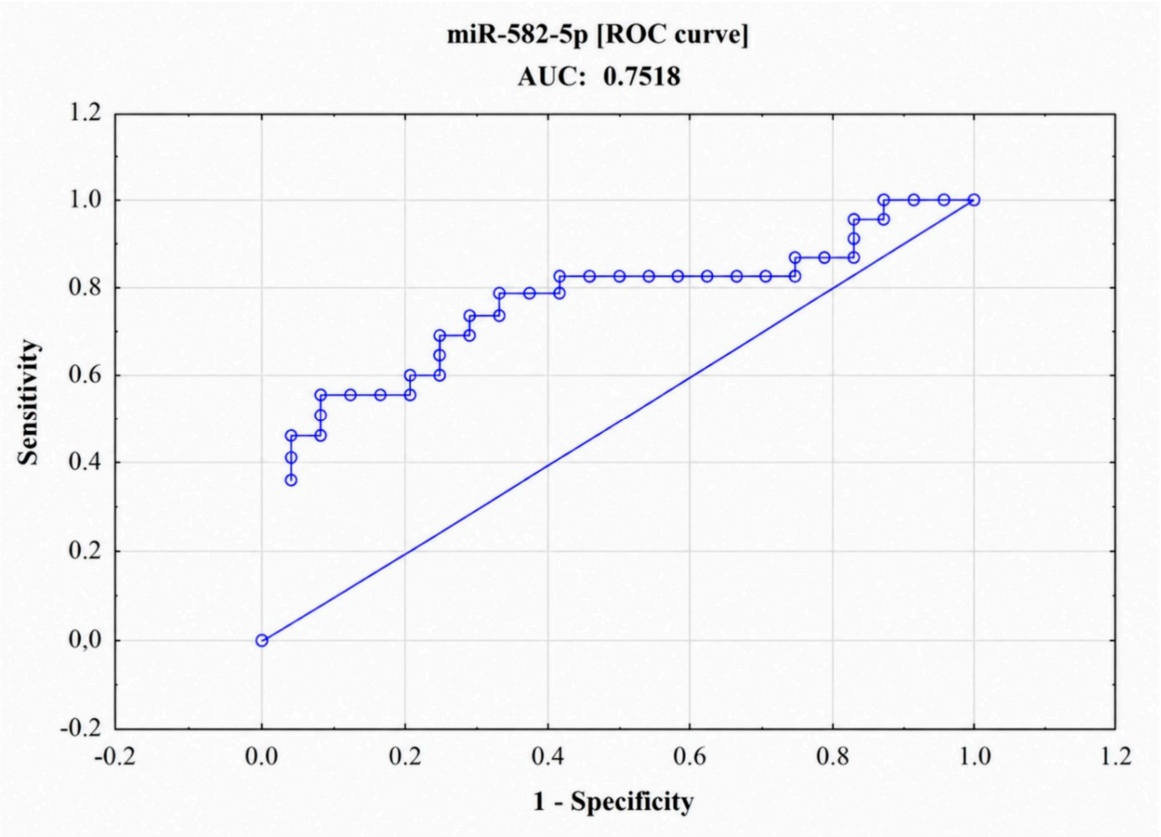

Figure S3. ROC curve for miR-381-3p (AUC=0.761)

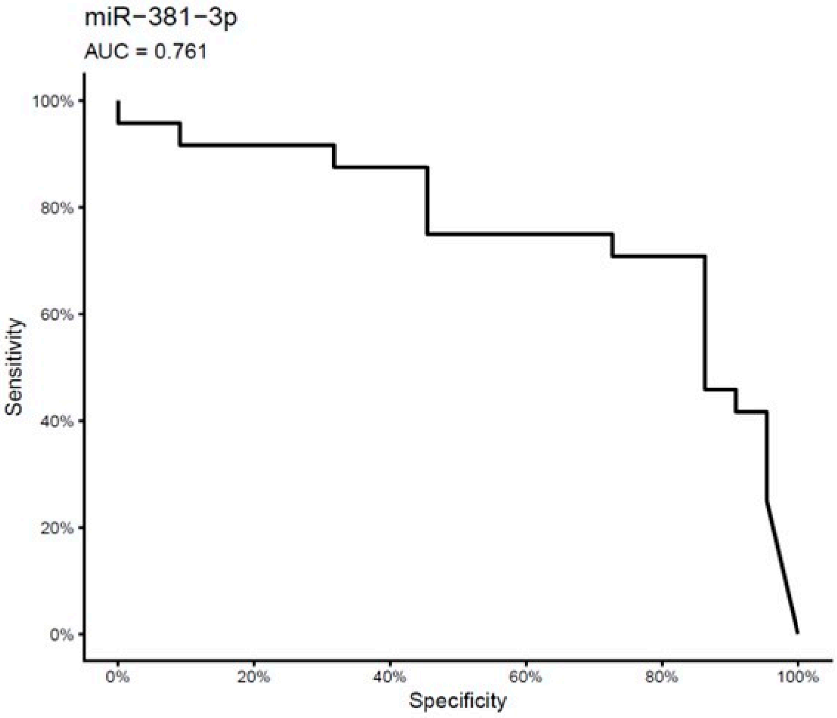

Figure S4. ROC curve for the exploratory three-miRNA panel (miR-425-5p, miR-381-3p, and miR-582-5p; apparent AUC=0.858).

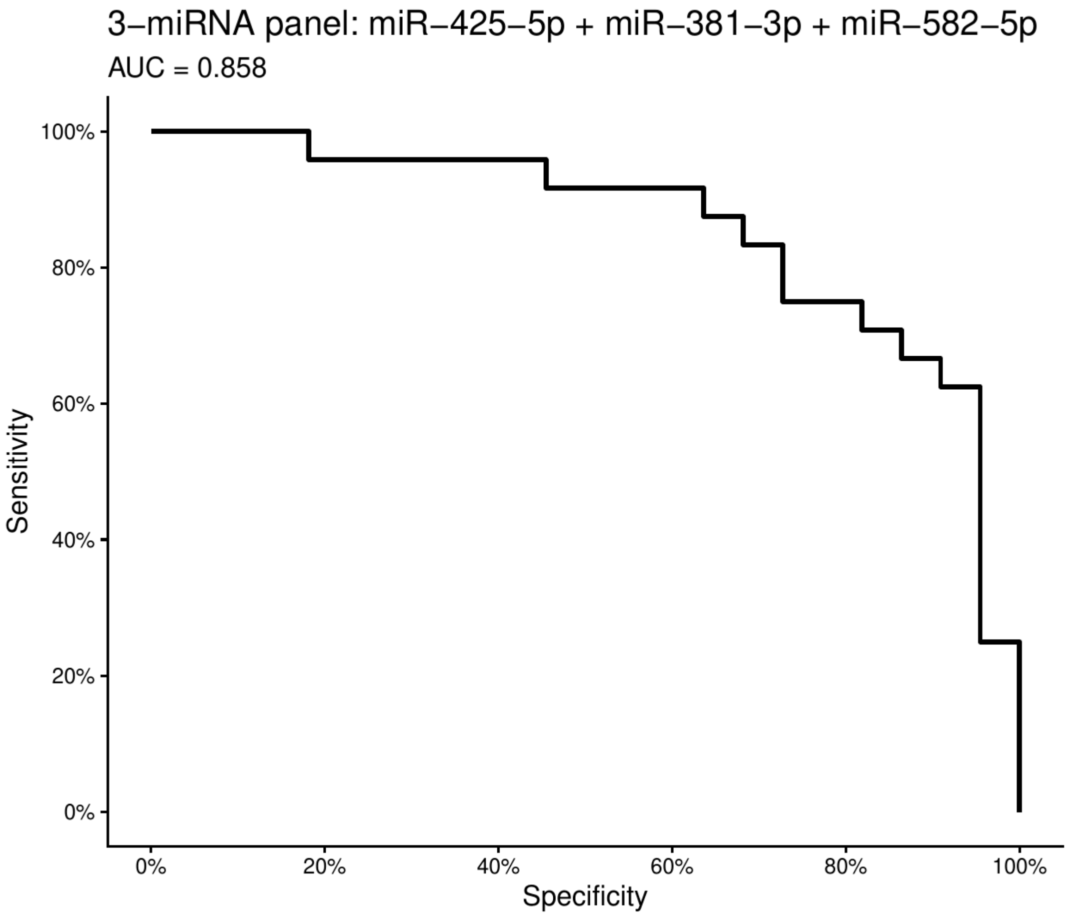

Figure S5. Kaplan-Meier curves for overall survival according to miRNA expression levels

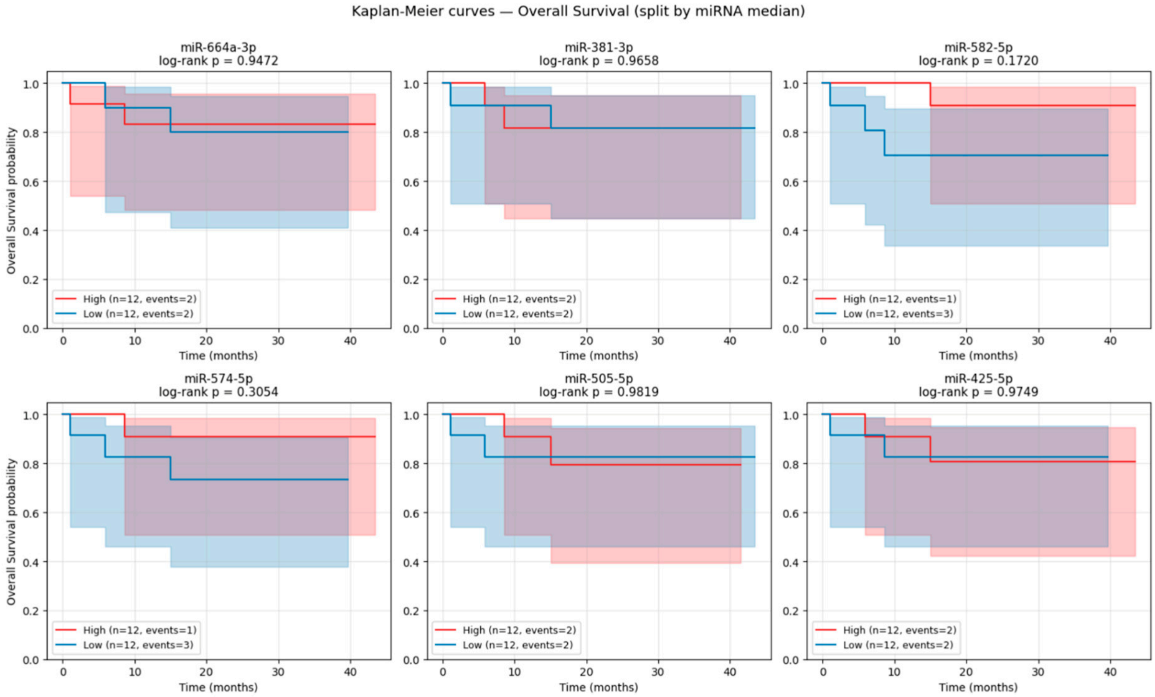

Figure S6. Kaplan-Meier curves for progression-free survival according to miRNA expression levels

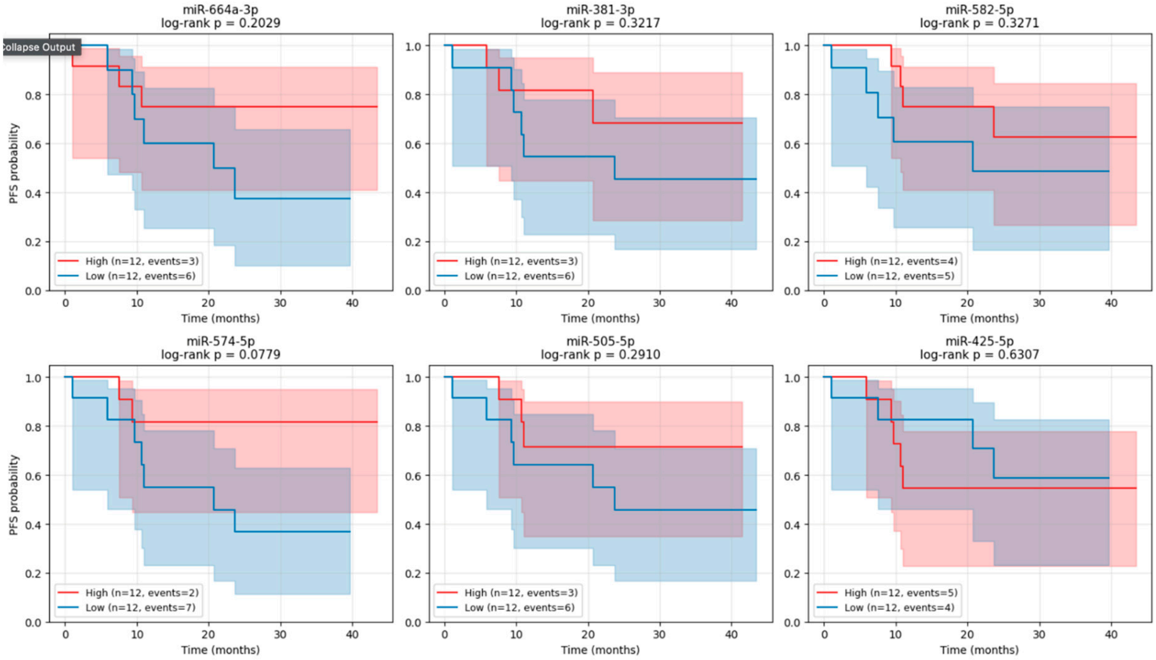

Figure S7. Correlation heatmap of analyzed miRNA expression levels in ovarian cancer patients

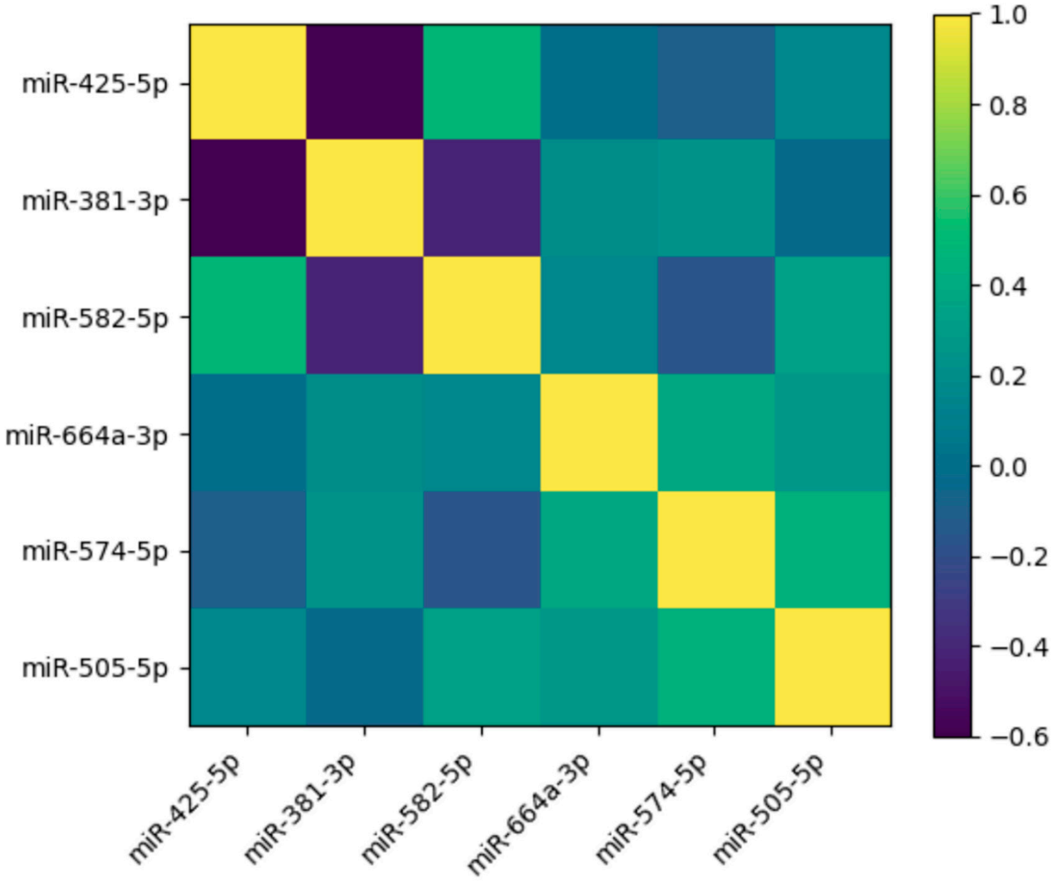

Supplement: Supplementary file 1 [file ijms-27-09122-s001.zip › ijms-4575969-supplementary.pdf]
